# Roach vs. Bonwill Clasps

**Published:** 1920-01

**Authors:** H. W. Allwine


### ROACH *vs.* BONWILL CLASPS

BY H. W. ALLWINE, D.D.S.

After a long experience with fixed bridge-work, the dental profession is trying to get away from much of it. The reasons are apparent to every dentist, and need not be repeated here. Dentoradiography is revealing to us hidden defective work that is a great cause of pyorrhea. To pyorrhea and other dental disorders are traced foci of infection of many bodily ailments. We want appliances that can be removed and that avoid the mutilation of teeth, and irritation of the soft tissues. We are returning to the partial plate with clasps.

I was much interested in an article in the October *Summary*, by Dr. Roach. We have all heard of Dr. Roach. We believe that he is giving us a beautiful substitute for some bridge-work. I believe, so far as use is concerned, it fills the want; I believe it is a thing of beauty, but the Doctor tells us it is not a joy forever. Let us see what the Doctor says:

"We believe that all clasps that do fit the tooth accurately, to be kept clean by the patient, must be left out at night; otherwise there is liability of damage following the employment of this type of clasp.

"I fully recognize that the clasp as we are making it today, that accurately fits the tooth and covers so much of the area of the tooth, is very liable to cause decay. It is a dangerous form of attachment to the natural teeth. If we do not impress upon our patients this danger and take the precaution to put this clasp in a condition that it can be kept clean and insist upon it being kept out at night injury follows. Impress it upon them that if they do not take care of them and take them out at night that the teeth surely will decay around them, and that if they do decay, it is their funeral and not yours."

The cast clasp fits with perfect coaptation. In this case, there is sure to be chemical action under the band. The Doctor tells us this, and we all know it. He would overcome this action under the band by leaving the appliance out at night. Is this enough? Allowing eight hours for rest, there are still left sixteen hours of the day in which time the plate is in the mouth. I can not see how this make of clasp can be long-lived; as it is sure to cause trouble.

I would make a suggestion. The main object of replacing teeth is to get proper mastication and insalivation. The Roach clasp and appliance are good. They fill the want while they last; hence they are of untold benefit to the patient. Why not leave the plate out at night and most of the day? Just leave it in enough during the day to prevent movement of the teeth so the appliance will not fit. The result, I believe, would justify this, and it would then be, so far as use is concerned, "a joy forever."

I would make one more suggestion. Crown the supporting teeth with properly-made crowns. In this way we get a permanent piece of work, so far as our work is considered, permanent. In crowning the tooth, all the bell should be taken off the tooth. A wire measurement at the neck of the tooth should slip off without being broken; this will give a close fit of the crown to the tooth. Before setting the crown it should be trimmed so that it will come barely to the gum margin, and bring the edge of the crown to a feather edge so it can be burnished to the tooth. Rather have the crown come short of the gum than go under it. Now put in the appliance, and it can remain all the time, having to be removed only to be cleansed. I would prefer doing this in the first place than to be forced to do it later.

I had the great pleasure of personal touch with Dr. Bonwill. We can all take off our hats to this great pioneer of dentistry. I not only read the Doctor's articles concerning the Bonwill clasp, but had the pleasure of seeing appliances in his mouth held by his own make of clasp. I also heard him go into detail as to how to make them. His clasp was made to touch the tooth at about four points only; he avoided perfect coaptation. He claimed that by thus avoiding a perfect fit, the motion of the soft tissues kept the secretions in motion under the clasp, and prevented decay that is sure to follow the cast clasp. He claimed two valuable points for his appliance—he avoided the mutilation of good teeth, and prevented the teeth from decaying under the clasps. I never heard him advise leaving the appliance out to avoid decay. I never do.

Do not take a bite and impression, send them to the laboratory, and expect a good fit. Make a good plaster cast of the teeth. Fit the clasps. Now put them onto the teeth. Make such changes as the case requires to adjust the clasp properly. Now, if you are making a rubber saddle, with the clasps in place, take a bite and impression and finish the case. If the base is of metal, fit the clasps, take an impression with the clasps and saddle in place; run cast in compound that will stand heat. Solder on the clasps. Now put to place in the mouth, take the bite and impression and finish case.

It will be remembered that in any of this kind of work, the only grinding of the teeth is to let the lugs up or down, as the case may be, to avoid contact with the occluding teeth.

The proper restoration of lost teeth requires physiological and anatomical knowledge, skill, good judgment, and care on the part of the operator. These requirements should be at hand with every dentist, whether he does the work or sends it to the laboratory. Laboratories often fail, not in point of beauty of execution, but in making what the case demands because they do not get the proper data from the dentist.—*Dental Summary.* .

